# Spatio-temporal patterns of the incoming water flow in pulsating corals

**DOI:** 10.1242/jeb.250262

**Published:** 2025-07-30

**Authors:** Dror Malul, Roi Holzman, Uri Shavit

**Affiliations:** ^1^ Faculty of Civil and Environmental Engineering, Technion IIT, Haifa 32000, Israel; ^2^ The Interuniversity Institute for Marine Sciences, PO Box 469, Eilat 88103, Israel; ^3^ School of Zoology, Faculty of Life Sciences, Tel Aviv University, Tel Aviv 69978, Israel

**Keywords:** Soft corals, Xeniidae, Mass transfer, Lagrangian analysis, Particle image velocimetry

## Abstract

Sessile marine organisms rely on water flows to enhance mass transfer and facilitate key physiological processes. Pulsating corals exhibit rhythmic pulsation of their tentacles to generate flow that removes excess oxygen produced during photosynthesis, thereby enhancing the process. The ejected flow is counterbalanced by an incoming flow directed toward the polyp, potentially delivering essential nutrients. This mechanism may be crucial for these corals, which rely on epidermal nutrient uptake rather than zooplankton predation. The characteristics of the incoming flow and its interactions with the coral tissue, where mass transfer occurs, are largely unknown. Here, we characterize the origin of new water approaching the polyp, the pathway it takes, where on the polyp and when during the pulsation period this interaction occurs. We used particle image velocimetry on single polyps to measure the flow field around the polyp and reconstruct the trajectories of the incoming water. We found that incoming water primarily originates from below the polyp. Eighty percent of the new water interacts with the polyp during the downward stroke of the tentacle motion, and 75% of the new water contacts the aboral face. We used a conservation of mass analysis to estimate the flow between the tentacles and found significant bidirectional flow through the gaps. Pulsation draws in ∼26,800 polyp volumes of new water daily, containing sufficient nitrogen to fully meet the polyp's estimated daily demand for growth. Increased nutrient uptake may explain the persistence of pulsation during the night when photosynthesis ceases.

## Teaser

**Summary:** Flows generated by pulsating corals draw in ∼26,800 polyp volumes daily, sourcing water primarily from below with substantial flow through tentacle gaps. These flows deliver sufficient nutrients to support growth.

## INTRODUCTION

Sessile marine organisms rely on mass transfer at the interface between their tissue and the surrounding water to facilitate essential biological processes such as respiration, photosynthesis, waste removal and nutrient uptake (Abelson et al., 1993; Kaandorp et al., 1996; Sebens et al., 1998; Finelli et al., 2006). The bidirectional fluxes of solutes between the water and the tissue are primarily driven by the concentration gradient across this interface. The fluxes are reduced when the gradient is diminished, e.g. when waste products accumulate in the water at the surface of the tissue, or when required nutrients are reduced there (Helmuth et al., 1997; Mass et al., 2010). In general, the concentration of solutes in the water surrounding the tissue *c* is described by the advection–diffusion equation, which describes the combined effects of advection by water flow and diffusion as follows:
(1)

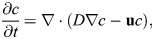
where *D* is the diffusion coefficient and **u** is the water velocity field. The augmentation of the water velocity **u** typically enhances advection from the surrounding environment to the region near the tissue, increasing the gradient at the tissue's interface, and potentially improving mass transfer.

Modifications of the ambient flow field can occur passively, without the organism actively moving, through morphological features that are advantageous for mass transfer, such as colony morphology and surface geometry. In branching corals, larger spacing between branches allows for greater flow penetration and higher mean velocities between the branches, resulting in enhanced mass transfer compared with tightly packed structures (Hossain and Staples, 2020). In massive corals, widely spread ridges increase turbulence near the tissue, whereas closely spaced ridges increase the total surface area available for diffusion, both beneficial for mass transfer (Stocking et al., 2018).

In addition to advantageous shape and surface geometry, sessile organisms can also utilize non-muscular motions, such as the movement of flexible appendages driven by water flow to alter the ambient flow around them. For example, coral tentacles have been shown to move out of phase with respect to ambient oscillatory flows, owing to their elastic properties. This motion increases the relative velocity between the tentacles and the surrounding flow, enhancing mass transfer by up to 25% compared with in-phase motion (Malul et al., 2020). Similarly, the flexible blades of aquatic vegetation sway with the water's motion, interacting with canopy-scale vortices. This interaction creates turbulence and enhances the exchange of mass between the canopy and the flow above it (Nepf, 2012).

Finally, sessile organisms can actively generate internal or external water flows that enhance advection and increases the rates of mass transfer. Filter feeders such as sponges (Larsen and Riisgåd, 1994; Morganti et al., 2021), ascidians (Riisgard, 1988) and bivalves (Jørgensen et al., 1986) use undulating cilia to create internal feeding currents, allowing them to pump water through their body for particle filtration. Barnacles create external feeding currents by repeatedly beating their cirral fans (Maar et al., 2024), and upside-down jellyfish by periodic pulsation of their bell margins (Santhanakrishnan et al., 2012). In filter feeders, the primary purpose of flow generation is particle capture; however, the enhancement of processes that depend on mass transfer, such as respiration, is an additional beneficial outcome of this mechanism.

**Fig. 1.**
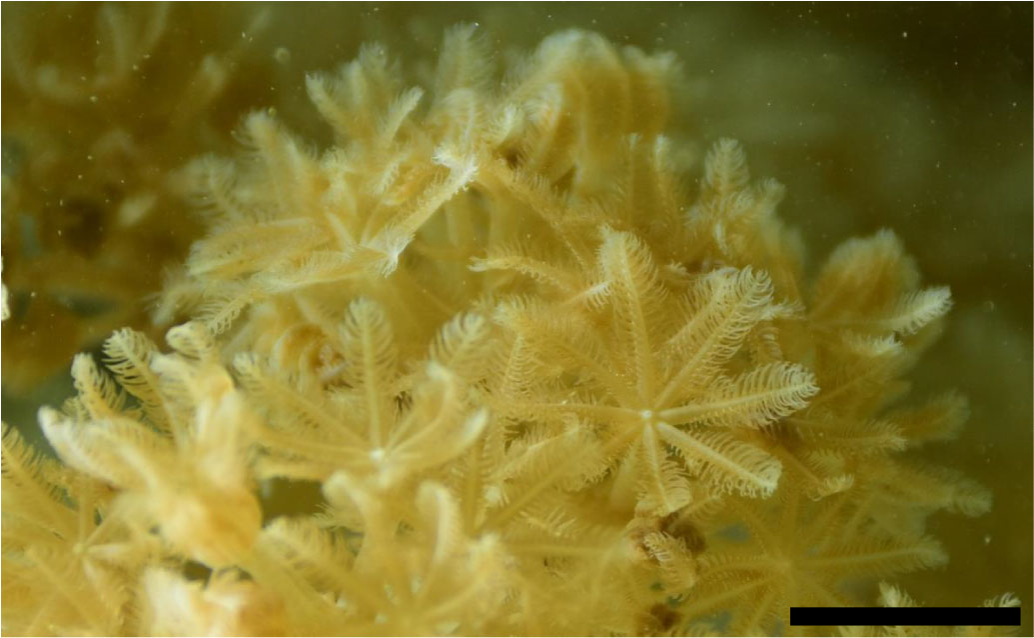
**A colony of *Xenia umbellata*.** Scale bar represents 1 cm.

Pulsating corals of the family Xeniidae (Fig. 1) use rhythmic pulsations of their eight tentacles to generate water flow (Kremien et al., 2013; Samson et al., 2019). These corals maintain a symbiotic relationship with algae that are hosted in specialized cavities in the coral's tissue. The coral supplies the algae with essential nutrients such as nitrogen and phosphorus, while the algae provide the coral with carbon through photosynthesis in return (Wiedenmann et al., 2023). Unlike other corals, which obtain their nutrient supply by preying on zooplankton, there is no evidence of predation in xeniid corals. Prey items are almost never found in their stomachs, nematocysts are sparse, and they are believed to primarily rely on absorbing dissolved materials from their surrounding environment to meet their nutrient needs (Goreau et al., 1971; Schlichter, 1982).

**Fig. 2.**
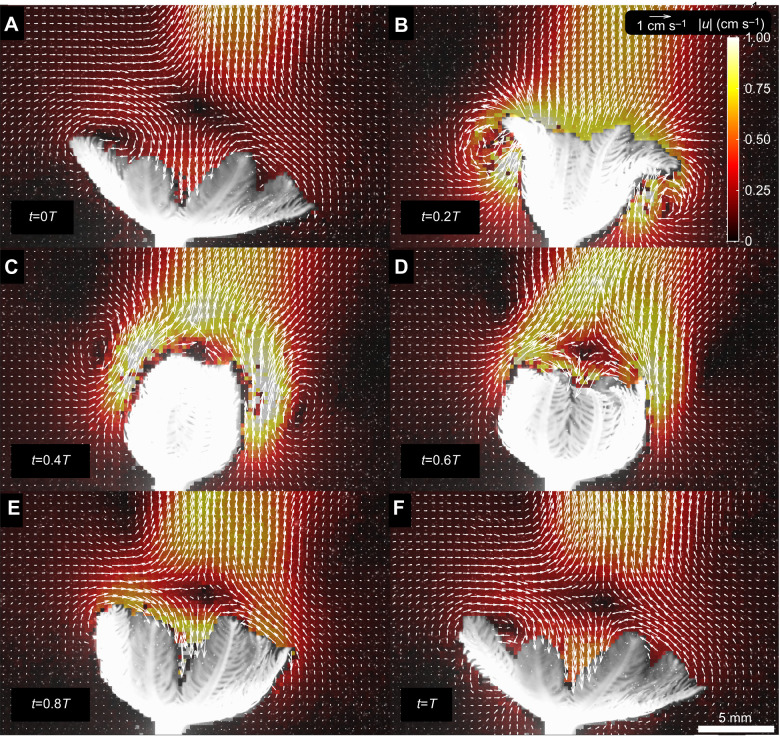
**Snapshots of the velocity field near a single polyp during one pulsation period.** Vectors represent point measurements of instantaneous water velocity, and the background color indicates velocity magnitude (a 1 cm s^−1^ reference vector and color bar shown in B). (A) The upward stroke begins with the tentacles spread out in a resting position, resembling the petals of a flower. (B,C) High velocities around the polyp are achieved during the upward stroke. (D–F) During the downward stroke, some water is drawn toward the polyp, while the jet directed away from the polyp is maintained. This example features a polyp with a diameter of 1.85 cm (scale provided in F), pulsating with a period of 1.95 s. Note that only a fourth of the measured vectors are shown.

Previous research (Kremien et al., 2013) demonstrated that pulsation in xeniid corals alleviates oxygen buildup near the tissue by removing excess oxygen, thereby enhancing photosynthesis rates by up to 10-fold. Building on this finding, Samson et al. (2019) devised a numerical solution of the flow field above polyps, supported by particle image velocimentry (PIV) measurements. They found an almost constant jet directed away from the polyp, effectively acting as a conveyor belt to carry waste products away from it. During the upward stroke, the contraction of tentacles (Fig. 2A,B,C) generates vertical water velocities of up to 1 cm s^–1^, feeding the jet. While the subsequent expansion of the tentacles (Fig. 2D,E,F) drives water towards the polyp, the flow of the jet above the polyp is maintained. Owing to conservation of mass, the outward flow from the polyp must be counterbalanced by an incoming flow. However, despite the potential importance of these incoming water to the polyp's nutrient uptake, the source of incoming water, its trajectories and interaction with the pulsating polyps are unclear. Additionally, our understanding of the dynamics of water flow through the (opening and closing) gaps between the tentacles is meager.

To understand the incoming flow and its interaction with the pulsating coral, we utilized high-resolution PIV to simultaneously measure the flow field around single polyps of *Xenia umbellata* and their kinematics. Our goals were to identify the direction from which new water arrives at the polyp, pinpoint the time and location where this water first intercepts the polyp, and quantify the flow rate through the gaps between the tentacles. Owing to the difficulty in directly measuring the flow between the tentacles, obstructed by the tissue, we applied integral mass conservation to estimate the flow rate through the gaps between tentacles throughout the pulsation cycle. Using the measured flow fields, we also performed Lagrangian analyses to study the timing and location of interception of new water with the polyp. Focusing on single polyps allowed us to examine the water approaching the polyp from beneath, an area typically obscured by neighboring polyps in a colony. This way, we elucidated the spatio-temporal patterns of the flow approaching the polyp and their interception with it, and the potential role of pulsation in delivering dissolved nutrients to the polyp.

List of symbols and abbreviations

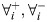
total volume entering (+) or exiting (–) the control volume during one pulsation period, where *i* refers to gaps or aperture
%N: nitrogen mass per dry mass of a polyp
∂Ω*_i_*: domain boundaries in the Lagrangian analyses, where *i* refers to top, bottom, side and polyp
*C*_0_: concentration of tracers entering the domain in the Lagrangian analyses
DM: dry mass of a polyp
*D*_polyp_: polyp diameter
*f*: frequency of pulsation
**l**: normalized location along the tentacle
*L*_t_: tentacle length
*M*_N_: estimated daily nitrogen requirement of a single polyp
*N*_pulsations_: number of pulsations in a movie
*Q*_aperture_: flow rate through the aperture of the polyp
*Q*_gaps_: flow rate through the gaps
*Q*_side_: flow rate through the side boundary of the control volume
*Q*_swept_: flow rate of the volume swept by the polyp
*Q*_up_: flow rate through the top boundary of the control volume
*Re*: Reynolds number of the flow through the top aperture of the polyp
*Re_f_*: bulk Reynolds number of the polyp
*t*: time
*T*: pulsation period
*T*_down_: duration of downward stroke
*T*_up_: duration of upward stroke
**u**: water velocity field
*u_r_*_,rel_: radial water velocity at the side boundary of the control volume relative to it
*u_r_*_,side_: radial water velocity at the side boundary of the control volume at the camera FoR
*U_r_*_,side_: radial velocity of the side boundary of the control volume
**U**_t_: velocity of the tentacle
*u_z_*_,rel_: axial water velocity at the top boundary of the control volume relative to it
*u_z_*_,up_: axial water velocity at the top boundary of the control volume at the camera FoR
*U_z_*_,up_: axial velocity of the top boundary of the control volume
*V*_CV_: volume encapsulated by the polyp
**X**: cylindrical coordinate system
**X**_0_: origin of coordinate system
**X**_t_: location along the tentacle
**X**_tip_: tentacle tip location
*Z*_up_: topmost location of the polyp mask
Δ*t*: time-step between two consecutive frames
η: leakiness
μ: growth factor of a polyp
υ,ξ: location of the tracers in the Lagrangian analyses
φ: phase during the pulsation period
Ω_domain_: domain in the Lagrangian analyses

## MATERIALS AND METHODS

### Study organisms

Colonies of *Xenia umbellata* Lamarck 1816 (Fig. 1) were collected from outdoor containers with continuously flowing seawater at the Interuniversity Institute for Marine Sciences in Eilat, Israel. Fragmentation to polyps was achieved by cutting individual polyps from mature colonies using stainless steel scissors and placing them, using a Pasteur pipette, on a bedding of spherical glass marbles. The polyps were left to recover in their holding tanks for at least a week until some of them naturally settled on the surface of the glass marbles. This procedure was conducted so that individual polyps could be transferred to an aquarium in the laboratory for PIV experiments, and to be able to manipulate their orientation.

### Experimental apparatus

Individual polyps (*N*=8; one video each) were filmed inside a glass aquarium (internal dimensions: 40×40×40 cm length×width×height) filled to a height of 38 cm. To minimize the boundary effect of the aquarium walls on the measured flow field, the polyps were positioned roughly at the center of the aquarium. Glass marbles with settled individual polyps were positioned on a Plasticine blob, functioning as a diarthrotic joint allowing the rotation of the marbles. The Plasticine was placed on top of a 19 LEGO^TM^ brick tower (dimensions: 7.8 mm length and width; 18.2 cm height). Materials were chosen due to their non-toxicity, and had no visible effect on the pulsation of the polyps. Polyps were kept in the aquarium for the duration of the experiment session, no longer than 5 h each time, and were returned to the outdoor containers. Seawater temperature in the laboratory experiments was similar (±1°C) to the surface sea water at the Gulf of Aqaba. Aquarium water was replaced between sessions.

### Particle image velocimetry

**Fig. 3.**
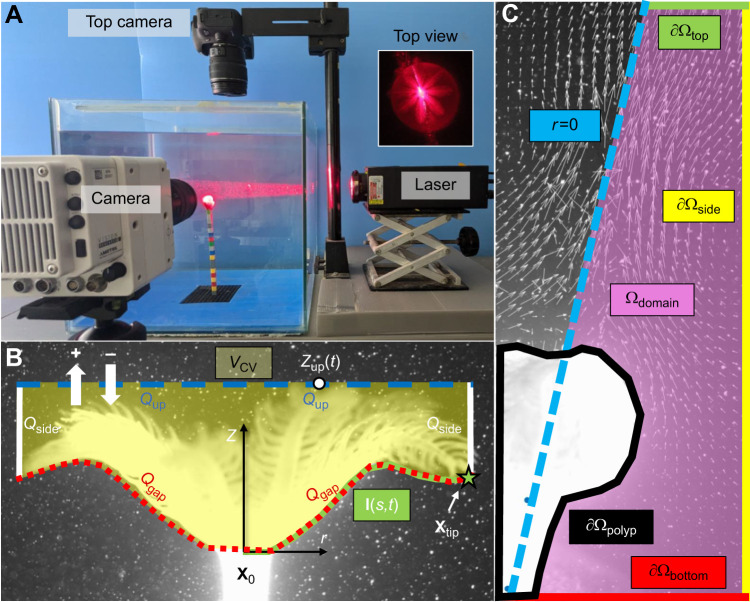
**Experimental setup, coordinate system and model definitions.** (A) PIV setup. Individual polyps were placed atop a LEGO^TM^ brick column (18.2 cm in height), centrally positioned in a 40×40×40 cm aquarium filled to a water height of 38 cm. A camera was positioned on the viewer side, with a laser light source placed at the side of the aquarium to create a laser light sheet (light red shading) in the camera's image plane. An additional camera was positioned above the aquarium to ensure proper alignment of the laser light sheet on the polyp (top view panel). (B) Definition of coordinate system, *V*_CV_, flow rates to/from *V*_CV_, and locations along the tentacle. The yellow shaded area denotes the projection of volume enclosed by the polyp in the *r*–*z* plane. The lines surrounding the yellow shaded area indicate where the flow rates to/from *V*_CV_ pass through: the dotted red line corresponds with *Q*_gaps_, solid white line with *Q*_side_, and the dashed blue line at the axial location *Z*_up_ with *Q*_up_. The origin at the mouth of the polyp is marked as **X**_0_ and the tip as **X**_tip_ (green star), and along the tentacle as **l**(*s*,*t*) (green line). (C) Definition of the domain and its boundaries in the numerical Lagrangian calculation. The numerical domain, Ω_domain_, is the pink shaded area bounded by the top (∂Ω_top_; green line), bottom (∂Ω_bottom_; red line) and right (∂Ω_side_; yellow line) boundaries. The *z*-axis (blue line) and the polyp (∂Ω_polyp_; black line) constitute the left boundary.

The PIV setup included a high-speed CMOS camera (Phantom VEO 340; Vision Research, Wayne, NJ, USA) with a resolution of 2506×1600 pixels, filming at 45 frames s^−1^. The camera was equipped with a 105 mm NIKKOR fixed-focus lens (Nikon Corporation, Tokyo, Japan), which provided a depth of view of ∼2 mm, an image plane width of 3.56±0.33 cm (mean±s.d.) and a height of 2.2±0.2 cm. The camera was aligned parallel to the floor to capture the polyp's side projection. A continuous wave laser (Coherent Inc., Santa Clara, CA, USA; 1.5 W, 685 nm) was positioned on the aquarium side. The laser's built-in optical system transformed the point laser to a laser light sheet (∼1 mm thickness) in the focal plane of the camera, which illuminated neutrally buoyant hollow glass spheres (10 µm diameter) suspended in the entire tank. The alignment of the laser on the polyp was adjusted and recorded using an additional camera (Canon EOS 7D Mark II; Canon Inc., Tokyo, Japan) with a Canon MP-E 65 mm f/2.8 5x Macro Lens, positioned above the aquarium to capture the polyp's top projection (Fig. 3A).

Eight videos were recorded, each featuring an individual polyp. Video duration ranged 15 to 33 full pulsation cycles (1390 to 3119 video frames). If polyps stopped pulsating or pulsated inconsistently, the video was cropped to include only the sections with repeatable pulsation patterns.

MatPIV 1.72 (Sveen, 2004 preprint) was used to resolve the water velocity vector fields. The PIV analyses were conducted in multi-pass mode with four passes applied. For the majority of the videos, we used interrogation windows of 128×128 and 64×64 pixels (each interrogation window twice). In two cases, we used smaller interrogation windows of 64×64 and 32×32 pixels. The spatial resolution of the interrogation windows was selected to maintain a vector rejection rate of ∼5%. Smaller windows were not used in all videos because they resulted in higher vector rejection rates in regions with locally low seeding density, leading to overall rejection rates exceeding 5%. An overlap of 50% between the interrogation windows was maintained in both scenarios. Consequently, individual vectors were spaced between 392 and 515 μm in the majority of the videos, whereas in the two cases with higher spatial resolution, the spacing ranged from 212 to 250 μm.

To mask the polyp and avoid erroneous vectors from high tissue reflectance, we used MiVOS (Cheng et al., 2021), a standalone, general-purpose interactive deep-learning tool designed and pre-trained for segmenting arbitrary objects in videos. The tool supports user input for corrections and was used without modification to efficiently isolate the polyp across frames and exclude high-reflectance areas from the flow field analysis. The segmentation output from MiVOS was validated frame by frame to ensure that the mask fully encompassed the polyp and excluded surrounding regions.

Data filtering was obtained by applying a signal-to-noise ratio filter (threshold 1.05), a global filter (s.d.=9) and a local filter (based on a 3×3 neighbors kernel; s.d.=2.5). On average, 5% of the vectors in each movie (range: 0.1–6.8%) were rejected and replaced using two-dimensional linear interpolation. At the end of every filming session, a snapshot of a ruler in the video's focal plane was used for calibration to physical dimensions.

Movie 1 provides an example of the flow fields obtained during a pulsation cycle.

### Phase averaging

Phase averaging was applied to all time-series signals we measured to reduce noise and obtain periodic signals representative of the polyp's pulsation behavior (Kim et al., 2013). Given a time-series signal, *f*(*t*), the phase-averaged signal, 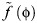, was computed by dividing the signal into pulsation periods, aligning them by phase, and calculating the median values at each phase point across all periods:
(2)

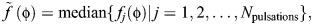
where *f_j_*(φ) represents the signal from the *j*th pulsation and *N*_pulsations_ is number of pulsations in a movie (see the List of Symbols and Abbreviations for a description of all symbols used in this study). Note that although the median was calculated, we will refer to this process as phase-averaging for convenience.

The spatially averaged velocity magnitude, 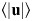 (where the norm bars signify absolute value and the angle brackets signify spatial averaging), was used as the reference signal. The time-stamps of local minima in this signal were assigned a zero phase, and the timestamps between these local minima were linearly assigned a phase up to a value of 2π (Fig. S1A,B). Incomplete periods of pulsation at the beginning and end of the video were cropped and not considered for the phase averaging. This technique was applied over the full length of the video. The resulting phased-averaged signals exhibited a narrow standard deviation, indicating consistent periodic behavior across cycles (Fig. S1C,D).

### Geometry and kinematics

#### Coordinate system

To take advantage of the axisymmetric morphology of the polyp and flow, we utilized a cylindrical coordinate system **X**=(*r*,θ,*z*). We set the origin *X*_0_ at the mouth of the polyp (Fig. 3B), and *z* to be orthogonal to the polyp's mouth. Based on a qualitative examination of the PIV movies, we assumed that the water velocity field, **u**=(*u_r_*,*u*_θ_,*u_z_*), had negligible swirl, therefore *u*_θ_ was assumed to be zero, therefore **u**=(*u_r_*,0,*u_z_*).

#### Tentacle location and velocity

Masking using MiVOS produced a binary mask of the polyp, which was also used to determine the time-dependent location of the tentacle visible in the camera frame. The algorithm generated a binary image for each frame, with pixel values corresponding to the presence (1) or absence (0) of the polyp. The boundary of the mask was traced using a custom MATLAB (MathWorks, Natick, MA, USA) script that employed the built-in function bwboundaries (Gonzalez, 2009), which detects the exterior boundaries and any interior holes of objects in a binary image. During manual validation of the MiVOS output, we ensured that the mask contained no holes and included only the polyp, so that bwboundaries would return a single boundary corresponding to the polyp.

The origin at the polyp's mouth (**X**_0_) was manually marked in each movie, and the time-dependent location of the tentacle tip, **X**_tip_(*t*)=(*R*_tip_(*t*),0,*Z*_tip_(*t*)) (green star in Fig. 3B), was defined as the farthest point of the mask relative to **X**_0_ on the right side of the polyp. The length of each polyp's tentacle, *L*_t_, was measured manually when the polyp tentacles were fully extended at the end of the downward stroke. The diameter of the polyp was estimated as *D*_polyp_=2*L*_t_. In addition to tracking the tentacle, we also tracked the axial location of the top of the mask, *Z*_up_ (white circle in Fig. 3B).

The location of the tentacle was defined as the line segment on the mask edge between **X**_0_ and **X**_tip_, marked as **X**_t_=(*r*_t_,0,*z*_t_,*t*). The velocity of the tentacle along its length is denoted as **U**_t_(*r*_t_,0,*z*_t_,*t*)=(*U*_t,*r*_,0,*U*_t,*z*_) where *U*_t,*r*_(*r*_t_,0,*z*_t_,*t*)=∂*r*_t_/∂*t* and *U*_t,*z*_(*r*_t_,0,*z*_t_,*t*)=∂*z*_t_/∂*t*. Numerical differentiation was obtained by the least squares method (Eqn S1). We defined a parameter 0<*s*<1 that notes the distance along **X**_t_, which satisfies **X**_t_(*s*=0)=**X**_0_ and **X**_t_(*s*=1)=**X**_tip_, and the normalized distance along **X**_t_ as **l**(*s*,*t*) (Fig. 3B).

#### Pulsation period

Fast Fourier transform (FFT) was used to determine the pulsation period. It was employed on 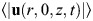 to obtain the frequency of pulsation, *f* (Frigo and Johnson, 1998). We compared the frequency obtained using a kinematic signal, namely the frequency of the *r*-wise location of the tentacle tip *R*_tip_(*t*), and the frequency obtained by a signal representative of the flow, 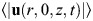, and found that both yielded almost identical results (linear regression: *R*^2^>0.99), suggesting that the flow field may be used to calculate the pulsation frequency independently from the kinematics. The period of pulsation was taken as *T*=1/*f*.

We classified the continuous motion of tentacles into two phases: the upward stroke with duration *T*_up_, and the downward stroke with duration *T*_down_ (*T*=*T*_up_+*T*_down_). The separation point was chosen as the time where the axial velocity of the midpoint of the tentacle, *U*_t,*z*_(*L*_t_/2,*t*), changed sign from negative to positive (Table S1A). To specifically investigate the natural variability and potential correlations between the pulsation period and tentacle length, we tracked 11 additional polyps exclusively for this analysis, bringing the total number of polyps analyzed to 19 (Fig. S1E).

### Mass conservation

To calculate the flow rate through the gaps between the tentacles, we utilized the integral equation of mass conservation for incompressible fluid:
(3)

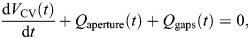
where *V*_CV_ is the volume enclosed by the polyp (yellow shaded area in Fig. 3B; see volume calculation in the Supplementary Materials and Methods and Fig. S2 for detailed description). *Q*_aperture_ is the flow rate through the top aperture of *V*_CV_, which is a sum of the flow rate through the top boundary (*Q*_up_; dashed blue line in Fig. 3B) and the flow rate through the sides of the boundary (*Q*_side_; solid white line in Fig. 3B). *Q*_gaps_ is the flow rate through the gaps between the tentacles (red dotted line in Fig. 3B). Note that the positive direction for the flow rates is out of the volume encapsulated by the polyp, and the negative is into it (white arrows in Fig. 3B).

The flow rate through the top boundary is calculated as the integral of the velocity across the surface area of the top boundary:
(4)

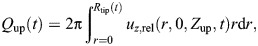
where *u*_*z*,rel_ is the axial water velocity at the top boundary relative to it, calculated as:
(5)

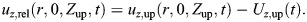
*U*_z,up_(*t*)=∂*Z*_up_/∂*t* is the axial velocity of the top boundary and *u_z_*_,up_(*r*,0,*Z*_up_,*t*) is the water velocity at the top boundary at the camera frame of reference (FoR).

The flow rate through the side boundary is:
(6)

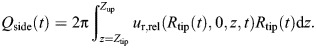
Note that during most of the pulsation period, *Z*_up_=*Z*_tip_, such that the top of the mask coincides with the height of the tentacle tip, and the side boundary is absent such that *Q*_side_=0. *u*_r,rel_ is the radial water velocity at the side boundary, calculated relative to the moving boundary as:
(7)

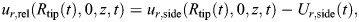
where *U*_*r*,side_=∂*R*_tip_/∂*t* is the radial velocity of the side boundary, and *u*_*r*,side_(*R*_tip_,0,*z*,*t*) is the water velocity at the side of the boundary at the camera FoR. By obtaining *Q*_aperture_, *V*_CV_ and its time derivative (using Eqn S1), we close the mass balance in Eqn 3 and obtain *Q*_gaps_.

We calculated the total volume of water exiting/entering *V*_CV_ during one pulsation period by integrating the flow rates over the duration of the pulsation cycle:
(8)

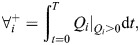

(9)

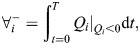
where subscript *i* may be replaced by gaps or aperture to represent where the flow rate being integrated, and superscript + indicates the volume that exits the *V*_CV_ and – indicates volume that enters *V*_CV_.

### Flow regime

To characterize the flow regime around aquatic animals, it is useful to use the Reynolds number (*Re*). Previous studies (Samson et al., 2019; Samson and Miller, 2020) used a bulk Reynolds number based on the frequency of pulsation and the tentacle length, defined as:
(10)

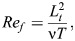
where ν=10^–6^ m^2^ s^−1^ is the kinematic viscosity of seawater.

To characterize the flow regime through the top aperture of the *V*_CV_, it is useful to use Reynolds' seminal formulation for flow through pipes (Reynolds, 1895):
(11)

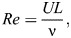
where *U* and *L* are characteristic water velocity and length scale. We take the diameter of the top aperture of the *V*_CV_, *D*_aperture_(*t*)=2*R*_tip_(*t*), as the characteristic length, and the characteristic velocity as the average time-dependent absolute velocity through the top aperture, calculated as *U*=|*Q*_aperture_(*t*)|/*A*_aperture_(*t*), where the area of the aperture is 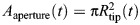, yielding 
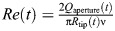
.

### Leakiness

Leakiness is defined as the ratio between the volume of fluid that moves through the gaps between the tentacles and the volume being swept by the tentacles motion (Cheer and Koehl, 1987). Here, we use flow rates instead of volumes to obtain the time-dependent leakiness:
(12)

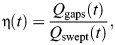
where:
(13)

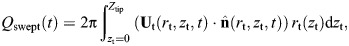
and 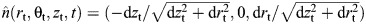 is a unit vector normal to the tentacle **X**_t_, oriented towards the control volume.

When 0<|η|<1, less volume leaks in between the tentacles than hypothetically being displaced by the tentacles and the gaps, and when |η|>1, more volume passes through the gaps than being hypothetically displaced by the envelope of the polyp. The sign of η indicates the direction of leakage: η>0 indicates that the direction of leakage and motion of the tentacles are the same, and η<0 indicates that the direction of leakage is opposite to the direction of motion.

### Lagrangian analysis

#### Numerical domain and boundaries

We used Lagrangian numerical calculations to study the source and destinations of water parcels around the polyp. That is, by simulating discrete tracer particles, we aimed to represent the continuous phenomenon of solute transport. The numerical domain, Ω_domain_, was defined as the flow field to the right of the polyp. The edge of the mask of the polyp was defined as its boundary ∂Ω_polyp_, and the top, bottom and side boundaries as ∂Ω_top_, ∂Ω_bottom_ and ∂Ω_side_, respectively (Fig. 3C; Table S1B).

Imaginary inertial-less tracers were introduced to the domain from its boundaries at a fixed concentration *C*_0_ and were allowed to move under the influence of the instantaneous measured water velocity. The tracers were removed from the simulation upon collision with the polyp or the boundaries of the domain. The original time-dependent velocity fields, rather than the phase-averaged ones, were used in this calculation, because the averaged flow fields tended to smooth out flow features such as vortex rings.

The sensitivity of tracer trajectories to the placement of domain boundaries was tested by artificially reducing the domain size and rerunning the simulations. We evaluated how the proportion of tracers reaching the polyp changed with decreasing domain width. When the distance between the right boundary and the rightmost tip of the tentacles exceeded one tentacle length, the effect on tracer interception was negligible. Therefore, only videos in which this distance was greater than one tentacle length were included in the analysis.

#### Imaginary tracers seeding

In each analysis, a total number of *N*_tracers_≈200,000 tracers were introduced to the domain boundaries at a fixed concentration *C*_0_, specified for each poylp. The concentration of tracers ranged from 218 to 1266 tracers cm^–3^ across polyps (Table S1B). During the simulation, for each volume of 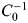 entering Ω_domain_ we have introduced a single tracer. If less than 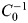 of water entered the domain during a given time step, the volume was accumulated along the next time steps, and only after a full water parcel was accumulated was a tracer seeded. See the Supplementary Materials and Methods for a detailed description of how *C*_0_ was determined.

#### Numerical calculation

Tracers were seeded with an initial location 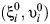 on the boundaries of the domain, where the subscript *i*∈{1…*N*_tracers_} denotes the index of the tracer. The time-dependent location, (*ξ*_*i*_(*t*), υ_*i*_(*t*)), was computed as:
(14)

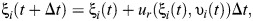

(15)

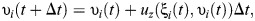
where Δ*t*=1/45 s is the time-step between two consecutive frames, and the velocity at (*ξ*_*i*_(*t*), υ_*i*_(*t*)) was linearly interpolated at any given time-step from the measured flow field using Delaunay triangulation.

At the beginning of the simulation, tracers were introduced from the domain boundaries and accumulated during a transient phase. This phase continued until the number of tracers entering the domain roughly equaled the number exiting it, indicating a quasi-steady state (Table S1B). The analysis of tracer movements and their interaction with the polyp was performed only after this state was reached, to ensure that the results reflected stable flow-driven transport dynamics rather than transient effects from initial conditions. To standardize the duration of all simulations, we artificially extended them to a total of 50 pulsation periods by appending the flow fields of 50–*N*_pulsations_ pulsation periods to the end of the original video (see Supplementary Materials and Methods for more details).

#### Interception of tracers

The tracers were classified based on their final location (Ω_domain_, ∂Ω_bottom_, ∂Ω_top_, ∂Ω_side_ or ∂Ω_polyp_), assuming collision with the polyp or the boundaries of the domain did not occur during the seeding phase. Tracers that collided with the polyp were further subclassified based on their destination, either the oral (top) or aboral (bottom) face. Focusing on single polyps allowed us to determine the specific locations on the aboral face and the timing of interception. However, owing to obstruction by the tentacles, we were only able to register the timing of tracers intercepting with the oral face, but not their exact location.

## RESULTS

### Kinematics and flow regime

We measured the length of tentacles and calculated their pulsation period and the durations of the upward and downward strokes. The length of tentacles ranged from 0.56 to 1.03 cm (mean±s.d.: 0.78±0.17 cm). The average pulsation period was *T*=2.26±0.42 s. *Re_f_* (Eqn 10) ranged between 9.8 and 54.5 s and averaged 29.6±14.4 s, covering the range of previously reported Reynolds numbers in pulsating corals [e.g. ∼10 in *X. umbellata* (Samson et al., 2019); ∼60 in *Heteroxenia fuscescens* (Kremien et al., 2013)]. Pulsations consisted of a relatively short upward stroke lasting *T*_up_=0.61±0.05 s and a longer downward stroke lasting *T*_down_=1.65±0.4 s (Fig. S1E).

Statistical analysis showed a moderate negative correlation between tentacle length and pulsation period (Pearson's correlation, *R*=–0.46, *P*<0.05). This trend is opposite to patterns observed in other pulsating marine organisms such as salps (Sutherland and Madin, 2010), squid (Gemmell et al., 2021) and jellyfish (McHenry and Jed, 2003), where larger individuals tend to pulse more slowly. However, the size range and sample size in our study are limited, and thus we are unable to draw general conclusions about scaling laws or typical behavior in *X. umbellata*.

**Fig. 4.**
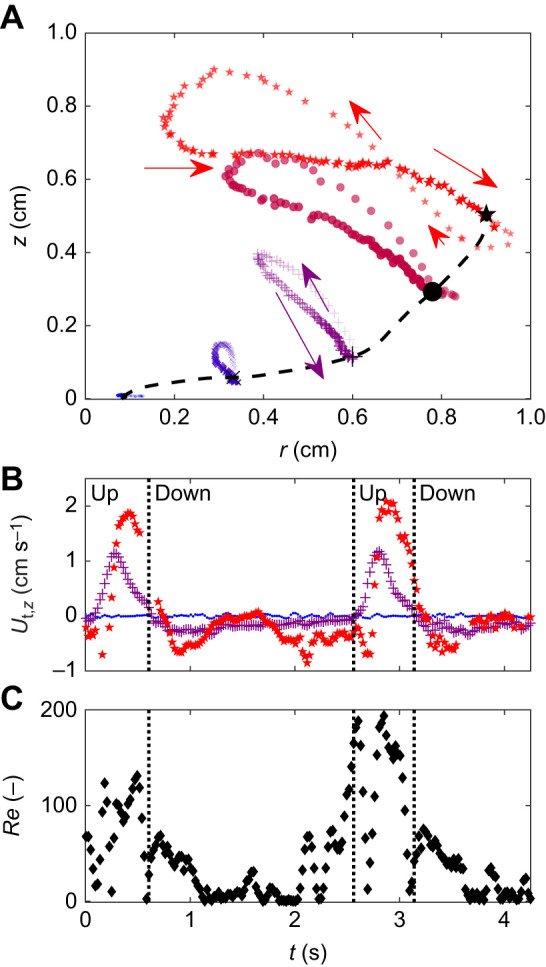
**A representative example of the kinematics of a tentacle and the *Re* number.** (A) Trajectories of five evenly spaced locations along the tentacle during one pulsation cycle. Each location is represented by a unique symbol and color ranging from blue (tentacle base) to red (tentacle tip). The transparency of the symbols indicates time where the most transparent symbols are at the beginning of the pulsation cycle. Arrows represent the direction of movement, where the smaller arrows are earlier in time. Black symbols and the black dashed line mark the location of the tentacle at the beginning of the pulsation period. (B) Axial tentacle velocity along the tentacle, *U*_t,*z*_(*s*,*t*). The colors and symbols are the same as in A. Dotted vertical lines delineate between the upward and downward strokes. The motion of the tentacle is led by the tentacle base (purple +), which is followed by the tip of the tentacle (red star). (C) *Re* number of the flow through the top aperture of the *V*_CV_ (Eqn 11).

We tracked the trajectories of landmarks along the tentacles in each polyp and observed their paths. Landmarks closer to the tentacle tip followed ∞-like trajectories, whereas those nearer to the base traced ellipse-like pathways (Fig. 4A). We calculated the time-dependent velocity of the landmarks along the tentacles and discovered that the axial velocity of the mid-point of the tentacle changed its sign from negative to positive 109±44 ms on average, before the tip of the tentacle did, indicating the start of contraction (one example in Fig. 4B). This suggests that the motion was driven by the base of the tentacle, with the distal half being passively dragged along, influenced by the base's movement.

The upward stroke was characterized by higher absolute velocities along the tentacle. At the tip of the tentacle, the average velocity during the upward stroke was 1.2±0.2 cm s^−1^, with a peak velocity of 2.2±0.2 cm s^−1^, compared with an average of 0.56±0.15 cm s^−1^ and a peak of 0.88±0.17 cm s^−1^ during the downward stroke. At the midpoint, the average velocity during the upward stroke was 0.5±0.06 cm s^−1^, with a peak of 1±0.2 cm s^−1^, whereas during the downward stroke the average was 0.23±0.04 cm s^−1^ and the peak was 0.37±0.07 cm s^−1^.

The higher tentacle velocities during the upward stroke resulted in substantially higher Reynolds numbers through the top aperture (Eqn 11; Fig. 4C). The average *Re* during the upward stroke was 42±20 (range: 20–77) and during the downward stroke 16±9 (range: 8–30). The maximal *Re* was 101±37 (range: 51–149) during the upward stroke, and 39±23 (range: 15–67) during the downward stroke.

### Flow rates

**Fig. 5.**
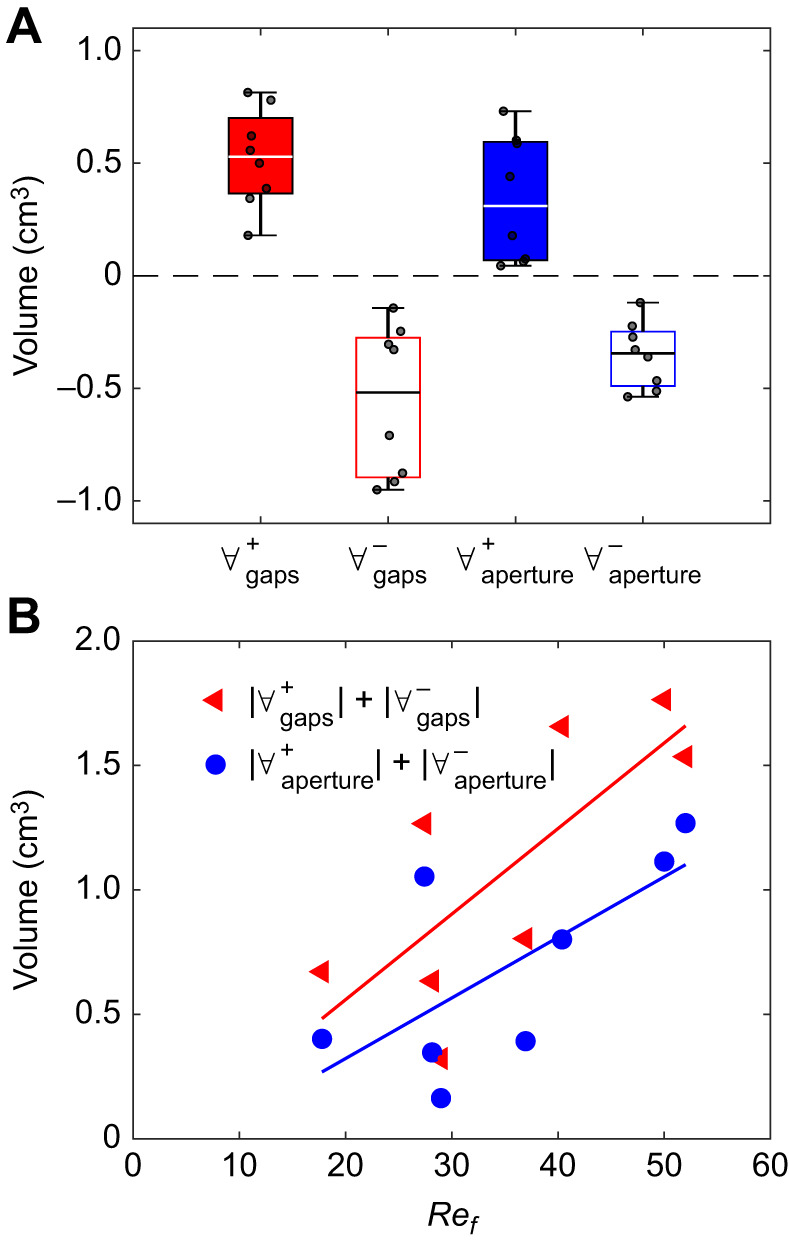
**Volume of water passing through the gaps (red) and the top aperture of the *V*_CV_ (blue).** (A) The filled bars represent water exiting *V*_CV_ (positive values; superscript +), and the outlined bars indicate water entering it (negative values; superscript –). The black scatter points show raw data for eight polyps. The horizontal line within each box marks the median, the box edges represent the first and third quartiles, and the whiskers extend to 1.5 times the interquartile range. (B) Relationship between the volume flowing through the gaps (red triangles) and through the top aperture of the *V*_CV_ (blue circles) with *Re_f_*. The lines show linear regression fits: 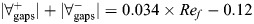 (*R*^2^=0.56, 95% CI for slope: 0.0042 to 0.064 cm^3^), and 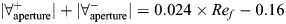 (*R*^2^=0.47, 95% CI for slope: −0.0012 to 0.05 cm^3^).

We measured the phase-averaged signals of the volume encapsulated by the polyp [*V*_CV_(*t*)] and the flow rate through the top aperture [*Q*_aperture_(*t*)] in eight polyps, and used Eqn 3 to estimate the flow rate through the gaps [*Q*_gaps_(*t*)]. The volume of water passing through the gaps in either direction during one pulsation period was 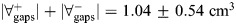 (median±s.d.), whereas the volume through the top aperture was 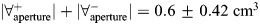 (Fig. 5A), indicating that the volume flowing through the gaps is comparable to the flow through the top aperture. We used a linear regression model to examine the relationship between 
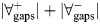
 and 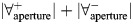 with *Re_f_* (Fig. 5B). Both 
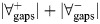
 and 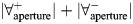 showed moderate correlations with *Re_f_*, with *R*^2^=0.56 and *R*^2^=0.47, respectively.

The extent of the flow through the gaps was also exemplified by the degree of leakiness of the polyps, calculated as a function of time throughout the pulsation period. In eight polyps, the average polyp leakiness was 0.9±0.23 (span: 0.45 and 1.24). Furthermore, during 45±13% of the pulsation period, the absolute value of leakiness was greater than one, indicating that the motion of tentacles augmented the flow through the gaps. This effect was greater during the upward stroke owing to the higher speed of the tentacle. The average absolute leakiness during the upward stroke was 1.74±0.52, roughly twice the value during the downward stroke, 0.77±0.4. During only 7±0.5% of the pulsation period the leakiness was lower than 0.1, a value previously estimated as the leakiness between tentacle cirri (Samson et al., 2019).

**Fig. 6.**
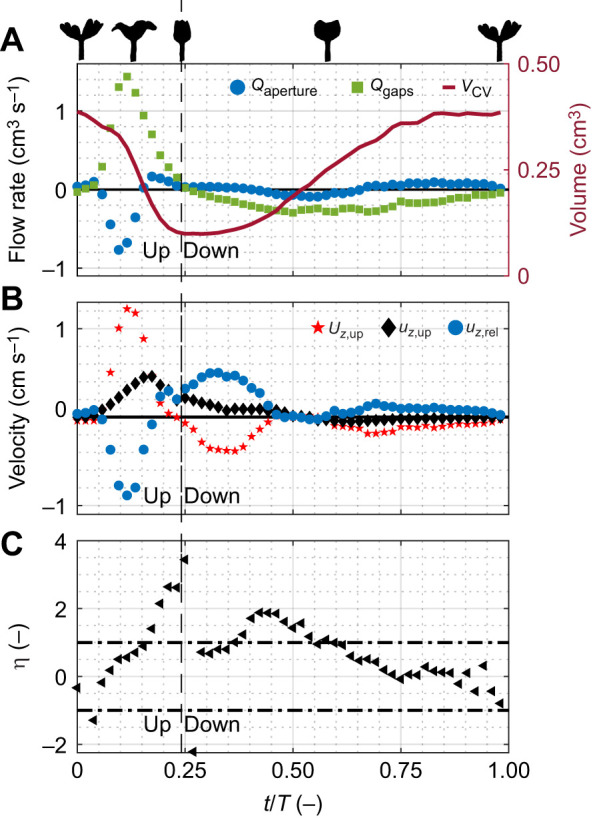
**An example of the temporal behavior of flow rates to and from *V*_CV_.** (A) *Q*_aperture_ (blue circles), *Q*_gaps_ (green squares) and *V*_CV_ (red line) during a representative phase-averaged pulsation period. (B) A comparison between the velocity of the top aperture (*U_z_*_,up_; red stars), the water velocity at the laboratory frame of reference (*u_z_*_,up_; black diamonds), and the water velocity at the polyp frame of reference (*u_z_*_,rel_; blue circles). The relative velocity (*u_z_*_,rel_) is calculated using Eqn 5 and serves as the basis for determining the flow rate at the top aperture shown in A. (C) Polyp leakiness, with horizontal dot-dashed lines marking |η|=1 for reference. Vertical dashed lines separate the upward and downward strokes. The example shows a polyp with *D*_polyp_=1.29 cm and a pulsation period of *T*=2.33 s (*T*_up_=0.56 s, *T*_down_=1.76 s; polyp 3 in Table S1A). The results represent the phase-averaged signal from 24 pulsation periods, with silhouettes provided above A for visual reference of the pulsation cycle. Sample density was halved to improve visualization.

Fig. 6 illustrates the dynamics of flow during a pulsation cycle, based on phase-averaged data (polyp 3 in Table S1A; see Fig. S3 for all eight polyps studied here). During the upward stroke, the volume encapsulated by the polyp decreases, with water flowing into it through its top aperture and out through the gaps. This finding suggests that the flow through the top aperture, driven by the change in the volume encapsulated by the polyp, has little to no contribution to the formation of the jet above it. The contribution of other possible drivers of the jet, such as momentum transfer owing to the motion of the tentacles, remains an open question warranting future investigation. During the downward stroke, the volume encapsulated by the polyp increases, with water inflowing primarily through the gaps and, to a lesser extent, through the top aperture. Flow rates are substantially higher during the upward stroke compared with the downward stroke, a trend that consistently appears across all eight polyps measured (Fig. S3).

When examining the flow fields in the laboratory frame of reference (Fig. 2A,B), the inward flow through the top aperture during the upward stroke may initially appear counterintuitive, as the velocity vectors above the aperture are directed upwards (*u_z_*_,up_; black diamonds in Fig. 6B). However, the flow through the top aperture of the polyp is measured in a moving frame of reference bound to the top of the polyp (Eqn 5). When the velocity of the tentacle (*U_z_*_,up_; red stars in Fig. 6B) is higher than the velocity of the water (*u_z_*_,up_; black diamonds in Fig. 6B), measured in the laboratory frame of reference, the result is a net inflow through the top aperture (*u_z_*_,rel_; blue circles in Fig. 6B). Also interesting is that during the last part of the pulsation period, the volume hardly changes and the inflow of water through the gaps is counterbalanced by an outflow through the aperture.

The average absolute leakiness in this example is 1.42 during the upward stroke, 0.71 during the downward stroke and 0.99 during the entire period (Fig. 6C). However, it is important to note that high leakiness values calculated between 0.2≤*t*/*T*≤0.25 and between 0.35≤*t*/*T*≤0.55 do not represent high flow rates, but rather lower values of the denominator in Eqn 12.

### Lagrangian analyses

#### Interception with the polyp

**Fig. 7.**
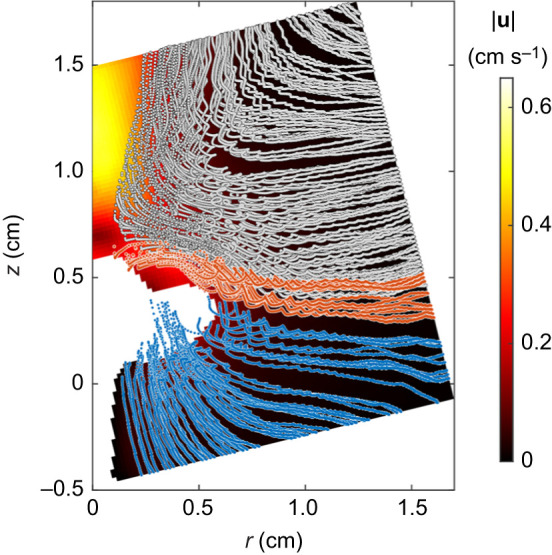
**An example of the tracer trajectories analysis.** The background color represents the temporally averaged flow velocity magnitude as shown in the color bar. The dotted points are the time-dependent locations of 200 randomly selected imaginary tracers is shown. For presentation, every third location of each tracer (every 66 ms) is shown. Tracer trajectories were colored according to their destination: blue, polyp aboral face; orange, polyp oral face; gray, exiting though the top of the domain. Note the spatial separation between tracers that reach the jet current and those that reach the polyp. The white background represents locations where the polyp, or part of it, was present ≥25% of the time. See polyp 8 in Table S1B for conditions in this specific Lagrangian analysis.

**Fig. 8.**
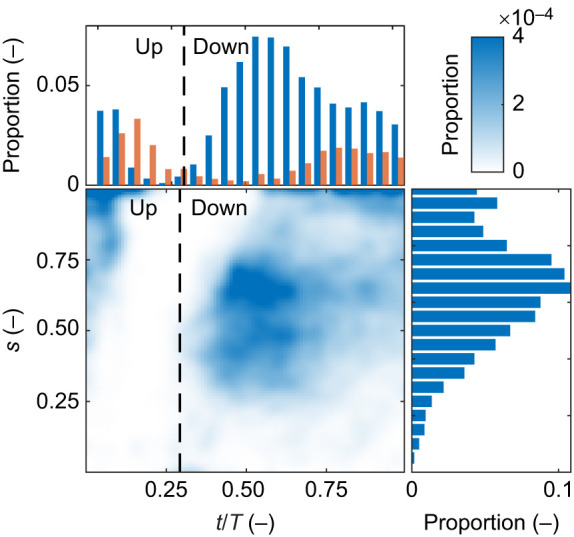
**Distribution of water parcel interception with the polyp.** The top panel shows the temporal distribution of tracers intercepted by the oral (orange) and aboral (blue) faces over an average pulsation period. The side panel illustrates the spatial distribution of interceptions along the tentacle length on the aboral face, which accounts for an average of 75% of all interceptions. The central panel displays the spatio-temporal distribution of interceptions on the aboral face, with the color bar located at the top right corner. Results are averaged across eight polyps, individual polyp data are presented in Figs S4 and S5.

We followed the trajectories of the traces entering the domain through its boundaries and measured the location and timing of tracer interceptions with the polyp in each PIV video. An example of typical trajectories observed in these analyses is presented in Fig. 7 and Movie S2. On average, the majority of interceptions (75±15%) occurred on the aboral face (Fig. S4), with 80±9% of all interceptions taking place during the downward stroke. The temporal distribution differed between the two faces: on the aboral face, 88% of interceptions occurred during the downward stroke, whereas on the oral face, 56% of interceptions occurred during the downward stroke (top panel in Fig. 8; Fig. S4). In total, 66% of all interceptions took place on the aboral face during the downward stroke, 9% on the aboral face during the upward stroke, 14% on the oral face during the downward stroke and 11% on the oral face during the upward stroke.

We calculated the cumulative proportion of tracers captured along the aboral face of the tentacle and found that, on average, 50% of them were intercepted by the distal part of the tentacle (side panel in Fig. 8; Fig. S4).

#### Angle of origin of tracers

**Fig. 9.**
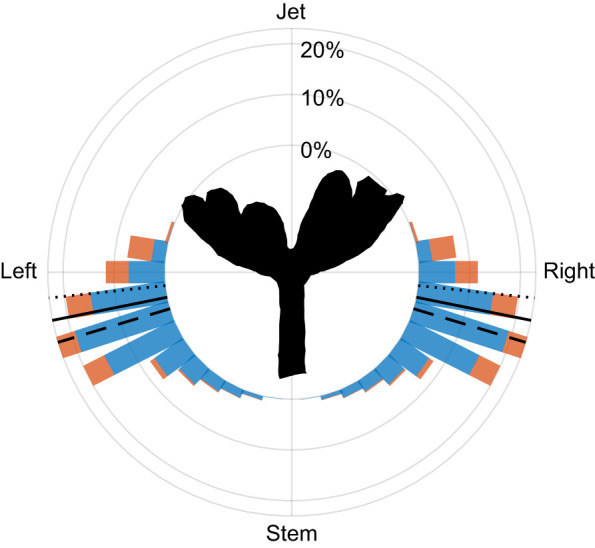
**Angle of origin of tracers reaching the aboral (blue) and oral (orange) faces of the polyp.** The histograms on the right show the average distributions from eight Lagrangian analyses, with angles measured relative to the polyp's mouth, represented by the black silhouette. Trajectories were only measured on the right side; the histograms are mirrored on the left side for visualization purposes. The solid line indicates the overall average angle of origin (–11±16 deg), the dotted line marks the average angle of tracers reaching the oral face –6±16 deg) and the dashed line indicates the average angle of tracers reaching the aboral face (–16±16 deg).

We measured the angle from which tracers approached the polyp relative to a coordinate system centered at the polyp's mouth. In this orientation, an angle of 0 deg represents tracers coming directly from the right side of the polyp, 90 deg represents those coming from directly above and –90 deg represents those coming from below. Tracers impacting the oral face approached from roughly the side of the polyp, averaging –6±16 deg, whereas those intercepted by the aboral face came more from beneath, with an average angle of –16±16 deg. The overall average angle of origin for all tracers was –11±16 deg (Fig. 9).

## DISCUSSION

In this study, we focused on the source of incoming water, its trajectories, the interactions with the pulsating polyp, and the rates of water flow through the gaps between the tentacles. We found that new water primarily arrives from beneath the polyp (Figs 7 and 9), as the aboral face of the polyp is responsible for ∼75% of the interaction with the new water (Fig. 8). Water arriving from underneath the polyp finds its way into the *V*_CV_ through the gaps between the tentacles (Fig. 5A). The dynamics of the exchange of water between *V*_CV_ and the surrounding flow are governed primarily by the motion of the tentacles (Fig. 6). Specifically, the downward motion of the tentacles, coupled with slow flows underneath the tentacles (Fig. 2E,F), causes water to move into the polyp's *V*_CV_ (Fig. 6A). Conservation of mass analysis showed that the flow rate through the gaps is similar in volume to the flow through the aperture during both the upstroke and downstroke (Fig. 5).

In the following sections, we will discuss the spatial and temporal patterns of the flow and interception of new water, the role of the gaps between the tentacles in facilitating water exchange, and the implications of our findings for colonies composed of multiple polyps. We also evaluate how pulsation-driven flow may enhance nutrient availability in the oligotrophic environment, which these corals inhabit.

### Spatio-temporal patterns

The flow generated by the motion of the tentacles can be separated into water that comes into contact with the polyp and water that bypasses it. Water that eventually reaches the aboral or oral face of the polyp (blue and orange trajectories in Fig. 7, respectively) arrives from below and the sides of the polyp (Fig. 9). This water enters the volume enclosed by the polyp *V*_CV_ either through the gaps between the tentacles (presumably making first contact with the aboral surface), or through the top aperture of the *V*_CV_ (presumably making first contact with the oral surface). However, a substantial amount of water is entrained by the jet (Nastase et al., 2008; green trajectories in Fig. 7) and is moved further away from the polyp without contacting it. Assuming that diffusion and dispersion processes are negligible relative to advection, water entrained in the jet appears to be ineffective for mass transfer and may therefore represent an energetic inefficiency, at least in the case of a single polyp.

During the short upward stroke, tentacle motion is relatively fast (Fig. 4B) and only ∼20% of the interceptions of new water occur. This suggests that the primary purpose of the upward stroke may be to expel water away from the polyp. Specifically, the fast motion contributes inertia that maintains an upwards flow away from the polyp in the form of the jet, persisting through both the upwards and downwards strokes.

The majority of the interception of new water occurs during the long downward stroke, when tentacle velocities are slower (Fig. 4B). The combination of high interception rates and longer residence time are consistent with increasing mass transfer rates, as previously suggested by Samson et al. (2019).

From comparing Fig. 5A and Figs 7 and 8, an apparent disagreement between the results of the Lagrangian and Eulerian frameworks may arise. According to the Eulerian framework (Fig. 5), the volume of water that enters *V*_CV_ from the aboral direction through the gaps (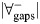) is similar to the volume entering from the oral direction through the top boundary of the *V*_CV_ (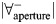). According to the Lagrangian framework, the rate of interception on the oral face is substantially lower than on the aboral side, where the tentacle gaps are (upper panel in 5; Movie S2). This apparent inconsistency is explained if the water passing through the top aperture of the *V*_CV_ largely consists of previously encountered water, depleted of tracers.

### Implications for colonies

Because we worked with single polyps, the implications for flow at the scale of a multi-polyp colony are presumed. In the natural environment, single polyps only exist at the early life stage of the coral. Single polyps begin budding within weeks after settlement (Nadir et al., 2023), eventually forming colonies with hundreds of polyps. The 3-dimensional flow field around these colonies is complex, with the flow around each polyp influenced by the flow generated by its neighbors, their mere presence and the ambient flow.

Focusing on single polyps simplifies some of the complexities present in colonies and offers the advantage of precise, high-resolution measurements of the flow field. This approach allows us to leverage the polyp's axisymmetry to infer the 3-dimensional flow field from 2-dimensional data. It also reduces the amount of water obscured by neighboring polyps, especially beneath the polyp, where flow data would otherwise be extremely challenging to obtain.

We postulate that the ability of a polyp within a colony to move water from below to the jet is lower than that of a single polyp. In colonies, neighboring polyps often have overlapping tentacles, which can restrict their range of motion and potentially reduce the volume of water transported. Even without tentacle overlap, adverse hydrodynamic interactions between adjacent polyps can occur. A computational fluid dynamics study (Samson and Miller, 2020) examined the effect of the spacing between two neighboring polyps and observed an ∼10% decrease in the average vertical velocity of water in their jet. Additionally, polyps within a colony may draw water expelled by neighboring polyps, limiting the inflow of new water. For these reasons, the single polyp case may represent an upper limit for the flow rate moved by individual polyps and the amount of new water interacting with them.

Despite the potential reductions in pumping performance of individual polyps within a colony, the water flow generated by them should be similar to that of a single polyp. Our observations of the kinematics of polyps within colonies further show qualitatively similar kinematics to those reported here. The average pulsation period we measured in 80 individual polyps from colonies was 1.89±0.1 s (our unpublished data), which is slightly shorter than the 2.26±0.42 s reported in this study. We therefore suggest that the flow patterns generated by the single polyp are relevant for understanding the general flow patterns through a colony. The general flow pattern of water drawn from beneath the polyp, passing through the gaps between the tentacles, and ultimately being expelled as a jet is expected to persist in colonies, even if the efficiency of individual polyps is slightly diminished.

In colonies, polyps are typically arranged in bouquet-like structures, with their oral surfaces facing upward and their aboral surfaces oriented toward the substrate (Fig. 1). This arrangement suggests a spatial separation of the volume around colonies, where water is drawn from below, and expelled to the freestream above it. *In situ* measurements of the flow field above colonies of *H. fuscescens*, another species of pulsating coral, show strong upward flows above the colonies (Kremien et al., 2013). These flows evacuate waste products into the freestream, preventing re-filtration of the used water by the colony. Water flow from beneath the colony offers a potential advantage in nutrient supply, as the water near the reef substrate is typically richer in nutrients compared with the freestream above (Richter et al., 2001).

### Functional role of the gaps

Our findings suggest that the gaps between the tentacles facilitate the refilling of the polyp volume. For example, during the downward stroke, when the volume encapsulated by the tentacles expands, the increase in volume must be balanced by flow through the top aperture or the gaps. Conservation of mass analysis indicates that inflow through the gaps (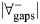) amounts to ∼20,100 polyp volumes per day (Figs 5A and 6A). This is comparable to the inflow through the top aperture, which is ∼13,800 polyp volumes per day. As shown in Figs 5A and 6A, a significant volume of water also exits through the gaps, mainly during the upstroke. The outflow through the gaps (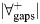; ∼21,200 polyp volumes per day) is comparable to the outflow through the top aperture (∼12,400 polyp volumes per day).

Such high volumes that pass through the gaps can be explained by their high leakiness compared with leakiness of structures in other organisms with similar beating behaviors. For example, Geierman and Emlet (2009) reported leakiness values of η<0.07 between the cirri of barnacles exposed to unidirectional flow velocities of 4 cm s^−1^, whereas Vo et al. (2018) found higher values of up to η=0.7 at increased ambient water velocities of 16 cm s^−1^. Using numerical simulations, Samson et al. (2019) estimated the leakiness between the thin pinnules extending from the tentacles of *H. fuscescens*, another species of pulsating coral, to be 0.1, suggesting that the slow flow between pinnules may allow for sufficient residence time for mass transfer to occur. The leakiness between the tentacles of *X. umbellata* polyps (present study) was consistently higher, averaging 1.74 during the upward stroke and 0.77 during the downward stroke, even in the absence of ambient flow. Hamlet et al. (2023) used numerical solutions of the flow field around the tentacles of gorgonian corals, which are morphologically similar to *X. umbellata* but do not pulsate, and estimated the leakiness between the tentacles to range between 0.7 and 1 with ambient flow of 2.5 cm s^−1^, directed perpendicular to the oral face of the polyp. Low leakiness values, as observed in barnacle cirri and the pinnules of pulsating corals, are particularly well suited for particle capture and enhancing mass transfer by reducing flow speeds and allowing particles or solutes to interact with the surface more effectively, whereas higher leakiness allows flow through the gaps. Our results show that leakiness values can exceed 1, which is uncommon in previous studies, where values typically fall between 0 and 1. In systems with stationary structures exposed to a free-stream flow, or with moving structures in a quiescent fluid, the volume of fluid passing between elements is constrained by either the ambient flow or the motion of the structures. As a result, leakiness in such systems is bounded by a maximum of 1. In contrast, the combination of active tentacle motion and self-induced flow in pulsing corals drives more fluid through the gaps than would be displaced by motion alone, resulting in leakiness values greater than 1.

The proposed role of the gaps between tentacles as an inlet for water depends on *Re_f_*. We observed a positive correlation between 
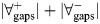
 and *Re_f_* (Fig. 5B). This can result from the effect of *Re_f_* on the thickness of the boundary layers on each of the tentacles, which reduces the average flow between them. Feitl et al. (2009) conducted an analysis of the boundary layers that develop around the lappets of ephyrae of scyphozoan jellyfish during beating. These ephyrae have a similar body design with eight axisymmetric lappets, exhibiting similar synchronous pulsating behavior. They estimated that the thickness of the boundary layer scales with 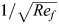. At low Reynolds numbers, the boundary layer becomes thicker, reducing the flow and leakiness through the gaps between lappets. They estimated that at the low range of *Re_f_* observed in their study (ephyrae with 1<*Re_f_* <10), the flow between the lappets is negligible, but as *Re_f_* increased beyond 10, the gaps would allow increasingly greater water flow.

### Potential for nutrient uptake

Most tropical corals rely on symbiosis with photosynthetic dinoflagellates hosted in their tissues to provide them with carbohydrates. These are supplemented with the capture of zooplankton prey, whose digested elements can be utilized by the host coral. Excess nutrients from the prey (organic nitrogen, phosphate and other trace elements) are thought to transfer to the photosynthetic symbiont, supporting their growth (Houlbrèque and Ferrier-Pagès, 2009). Unlike many other corals, evidence suggests that pulsating corals do not prey on zooplankton predation. Xeniid corals have been shown to absorb nutrients by epidermal uptake (Schlichter, 1982), and it is hypothesized that nutrient supply is compensated in this way. Therefore, we ask what is the potential transfer rate of nutrients to the polyps owing to pumping, and whether this rate is sufficient to support the growth of the coral and its symbionts.

Using nitrogen as a test case, the potential nutrient transfer can be estimated by taking the daily volume of new water that passes through the polyp and multiplying it by the ambient concentration of dissolved nitrogenous nutrients present on the reef. We compare these estimates with the daily nitrogen requirement of one polyp *M*_N_ as:
(16)

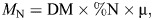
where DM=5.85±0.6 mg is the dry mass of a single polyp, measured by drying 52 individual polyps in uneven batches ranging from 1 to 20 polyps per weighing dish at 60°C for 24 h in a drying oven (DHG-9240A; YIHENG, Shanghai, China) and %N=0.05 is the nitrogen mass per dry tissue mass (Hill et al., 2023). The growth factor, μ, is derived from the exponential of the specific growth rate (SGR) as μ=1–*e*^SGR^. It represents the rate of polyp mass added per day, with values ranging from μ=0.02 to 0.05 day^–1^ (fig. 2 in Tilstra et al., 2023). Consequently, the estimated daily nitrogen demand of a single polyp ranges between 5.85 and 14.63 μg N polyp^−1^ day^−1^, or 1 and 2.5 mg N g^−1^ DM day^−1^.

The annual mean concentration of dissolved inorganic nitrogen (DIN; NO_3_^–^+NO_2_^–^+NH_4_^+^), measured monthly in 2023 at eight coastal locations along the Israeli coast of the Gulf of Aqaba, is 0.25 μmol l^−1^ (Shaked and Genin, 2023). At that concentration, a single polyp would need to completely absorb the nitrogen contained in 1.67 to 4.18 l of seawater daily to meet its estimated nitrogen demand for growth.

We estimated the median inflow of new water through the gaps (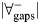) to be 20.1 l day^−1^. Assuming this flow represents 75% of all new water approaching the polyp (with the rest entering through the top aperture), the total daily volume of new water interacting with the polyp is 26.8 l day^−1^. Taking into account the variance between the flow observed for individual polyps, the flow through them ranged from 9.9 to 53.3 l day^−1^, indicating that the volume of water drawn daily through pulsation contains at least twice the polyp's estimated nitrogen demand.

To fully satisfy the nitrogen demand of the polyp, the required uptake efficiency should range from 3% at the lower bound to 42% at the upper bound, depending on the combination of nitrogen demand and the volume of water drawn. To the best of our knowledge, there are no direct measurements of nitrogen uptake efficiency in pulsating corals. However, a study by Schlichter (1982) offers a potential point of reference. In that study, radioactively labeled amino acids were used to measure uptake rates of 12 amino acids in the pulsating coral *H. fuscescens*. We used these reported uptake rates to estimate uptake efficiency, calculated as the ratio between the measured uptake rate and the incoming flux of each amino acid. The incoming flux was estimated as the product of the median water flow rate measured in the present study (26.8 l day^−1^) and the ambient concentrations used in Schlichter's experiments. The resulting uptake efficiencies ranged from 11% to 62%, with an average of 30%.

DIN, particularly ammonium, is generally considered more readily available and more efficiently assimilated by corals than dissolved organic nitrogen such as amino acids. This is reflected in consistently higher assimilation rates of DIN across coral taxa (Pupier et al., 2021). As such, the uptake efficiencies we estimated based on amino acid assimilation likely represent a conservative lower bound for DIN uptake efficiency. Ammonium uptake rates reported for corals provide useful benchmarks for evaluating the feasibility of our estimated requirements. In particular, Den Haan et al. (2016) reported an ammonium uptake rate of approximately 1.8 mg N g^−1^ DM day^−1^ for a hard coral species, a value closely matching the range estimated in our study (1 to 2.5 mg N g^−1^ DM day^−1^).

These estimates lend support to the idea that pulsation might allow a substantial transfer of nutrients to the polyps, a mechanism that (to the best of our knowledge) was not previously suggested. Pulsation was previously shown to augment photosynthesis and (to a lesser extent) respiration (Kremien et al., 2013; Mezger et al., 2022); however, these are not necessarily the growth-limiting rates for corals at the reef (Wiedenmann et al., 2023). The idea that large metazoans utilize nutrients through epidermal uptake is often controversial because of the limited effectiveness of diffusion for large organisms. Here, we show that a continuous flow of water could bring a substantial amount of nutrients, potentially fulfilling the polyp's needs. Admittedly, we based our calculation on typical values of growth, nutrient concentrations and flow through the polyps, all of which have considerable variation. We also based our estimation on DIN concentrations in the water column, whereas nutrient concentrations near the substrate may be higher (Richter et al., 2001). Although verifying the hypothesis requires experimental work (e.g. labeling of nutrients with stable isotopes), our results indicate that the mechanism is plausible, at least in terms of the rates of transfer of nutrients. Ambient water flow likely complements this mechanism, with pulsation being especially critical where and when ambient flow is low. The ability to absorb nutrients may also explain the persistence of pulsation during the night, when photosynthesis ceases, as nutrient uptake is independent of light availability.

## Supplementary Material

10.1242/jexbio.250262_sup1 Supplementary information
