# Supplementary material for: Spatio-temporal patterns of the incoming water flow in pulsating corals
Source: J Exp Biol. 2025 Jul 30;228(15):jeb250262. doi: 10.1242/jeb.250262 (PMC12377810; doi:10.1242/jeb.250262)
Supplement: Supplementary information [file jexbio-228-250262-s1.pdf]

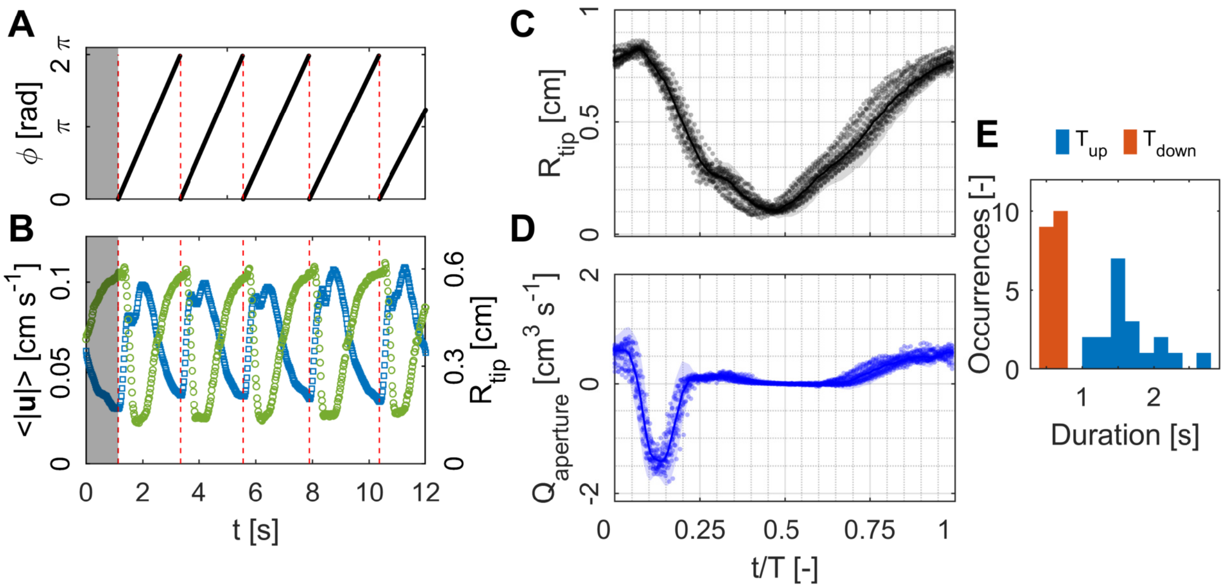

Fig. S1. Phase Averaging and distribution of stroke durations. (A, B) An example of the assignment of phase (ϕ) to individual timestamps is shown in A, using the spatially averaged flow velocity magnitude signal depicted in B (blue circles). For reference, the location of the tip of the tentacle is also shown in B on the right y-axis (green circles). Zero phase was assigned to timestamps in A where $|\boldsymbol{u}|$ reaches a minimum, with phases linearly distributed between 0 and 2π in between consecutive minima. The time prior to the first zero phase was excluded from phase averaging (gray shading). (C,D) Phase-averaged signal and corresponding raw data. Faded dots represent the raw data of 21 periods with the assigned phase, while the solid lines represent the phase-averaged signal as computed using Eqn 2, and the shaded background denotes two standard deviations from the median. An example of the kinematics is given in C as the radial location of the tentacle tip (R_{tip}), and the flow rate through the top aperture of the V_{CV} ($Q_{aperture}$) is exemplified in D. In this example $T = 2.24\ s$ and $D_{polyp} = 1.82\ cm$ (polyp 3 in Table S1A). (E) Distribution of the duration of the upward (T_{up}) and downward (T_{down}) strokes in 19 polyps. The mean T_{up} is 0.61 s ($s.d. = 0.05\ s$), and the mean T_{down} is 1.65 s ($s.d. = 0.4\ s$).

Supplementary Materials and Methods

Numerical differentiation

Numerical differentiation was performed using the least squares method. Given a set of discrete function values f_i at points x_i, the derivative is,

$$\frac{\partial f}{\partial x} \approx \frac{2f_{i+2} + f_{i+1} - f_{i-1} - 2f_{i-2}}{10\Delta x} \tag{S1}$$

This method is designed to minimize measurement uncertainly, which makes it ideal for experimental data, however it may smooth out the estimate of the derivative at peaks due to the greater weight it grants the outer points.

Calculation of V_{CV}

We estimated V_{CV} by calculating the volume of revolution of the tentacle. Most of the time the curve describing the tentacle position was a one-to-one function (Fig. S2A), meaning that for every z_t on the curve there was only one r_t value, then the volume was calculated as,

$$V^I_{CV}(t) = \pi \int_{z_t=0}^{Z_{tip}} r_t(z,t)^2 dz_t \tag{S2}$$

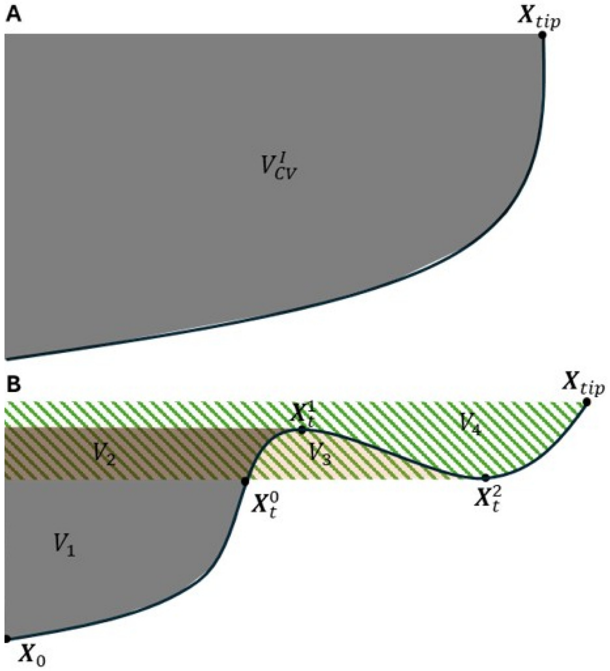

Fig. S2. Calculation of V_{CV}. (A) The area of V_{CV} is projected on the r-z plane, assuming the curve describing the polyp is a one-to-one function. V_{CV} is calculated using Eqn S2. (B) When the curve describing the tentacle is not a one-to-one function, calculating V_{CV} with Eqn S2 will incorrectly include the additional volume V_2 twice and V_3, which are not part of the actual volume. The calculation in Eqn S3 accounts for this and provides the correct volume.

At other times, such as at the beginning of the upward stroke when the distal half of the tentacle curved downward, the function describing the tentacle was no longer one-to-one. As a result, for certain values of z_t there could be up to three corresponding z_t values. During these

Journal of Experimental Biology • Supplementary information

times, a piece-wise calculation of the volume has to be done in order to avoid accounting for the same volume more than once. Figure S2B shows an example of a situation where a piece-wise calculation is required. When integrating along the tentacle between $\boldsymbol{X}_0$ and $\boldsymbol{X}_{\text{tip}}$, the volume marked as V_2 is counted three times and therefore should be subtracted twice from the final result. Additionally, the volume marked as V_3 is erroneously accounted for once where it should not be, so it must also be subtracted from the final result. We mark the two critical points on the tentacle, $\boldsymbol{X}_t^1 = (R_t^1, 0, Z_t^1)$ and $\boldsymbol{X}_t^2 = (R_t^2, 0, Z_t^2)$ and another point $\boldsymbol{X}_t^0 = (R_t^0, 0, Z_t^0)$, which has the same z_t coordinate as $\boldsymbol{X}_t^2$. These points define the sections used in the piece-wise calculation, where we integrate along the length of the tentacle and subtract the overlapping volumes, which results in the following expression,

$$V_{CV}(t)^{II} = V_{CV}^{I}(t) - \pi \int_{z_t=Z_t^0}^{Z_t^1} r_t(z_t,t)^2 dz_t - \pi \int_{z_t=Z_t^1}^{Z_t^2} r_t^2(z_t,t) dz_t \tag{S3}$$

Additionally, at times due to imperfections in the symmetrical pulsation of the polyp, one of the other tentacles bulged above the tip of the tentacles used for tracking (such as the case show in Fig. 3B). In these cases, the volume up to the topmost location of the mask ($\boldsymbol{X}_{up} = (R_{up}, 0, Z_{up})$; white dotted line in Fig. 3B) was also accounted for,

$$V_{CV}(t)^{III} = V_{CV}^{II}(t) + \pi R_{tip}^2 (Z_{up} - Z_{tip}) \tag{S4}$$

Concentration of tracers in the Lagrangian simulations

To determine the required concentration of tracers, C_0, we measured the total volume of water entering the domain during each movie, $\forall^{total}$, by integrating the time-dependent inward flow rate of water that enters the boundaries of the domain ($\partial\Omega_{bottom}$, $\partial\Omega_{side}$, and $\partial\Omega_{top}$) and over the duration of the movie t_{end}:

$$\forall^{total} = -2\pi \overbrace{\int_{t=0}^{t_{end}} \int_{\partial\Omega_{side}(u_r\langle 0)} r u_r dz dt}^{\partial\Omega_{side}} + 2\pi \overbrace{\int_{t=0}^{t_{end}} \int_{\partial\Omega_{bottom}(u_z\rangle 0)} r u_z dr dt}^{\partial\Omega_{bottom}}$$
$$\text{-}2\pi \overbrace{\int_{t=0}^{t_{end}} \int_{\partial\Omega_{top}(u_z\langle 0)} r u_z dr dt}^{\partial\Omega_{top}} \tag{S5}$$

Extension of Lagrangian simulations

We aimed to minimize the Root Mean Square Error (RMSE) between the flow field of the last frame in the original video and the first frame of the appended frame. The first frame was chosen as the first frame which had a local minimum in the RMSE, hence being most similar to the last frame of the original video.

$$\epsilon_m = \sqrt{\frac{1}{HW}\sum_{a,b}(u_r^{a,b,c} - u_r^{a,b,N_0})^2 + \frac{1}{HW}\sum_{a,b}(u_z^{a,b,c} - u_z^{a,b,N_0})^2} \tag{S6}$$

Journal of Experimental Biology • Supplementary information

where ϵ_m is the RMSE between the last frame N_0 and the mth frame. $a \in \{1, 2, .., H\}$ and $b \in \{1, 2, .., W\}$ indicate the instantaneous flow velocity measurement from the PIV analysis, where H and W are the number of measurements in the vertical and horizontal directions. So that the entire expression for ϵ_m is the average RMSE between the flow velocity in the N_0 and mth frame.

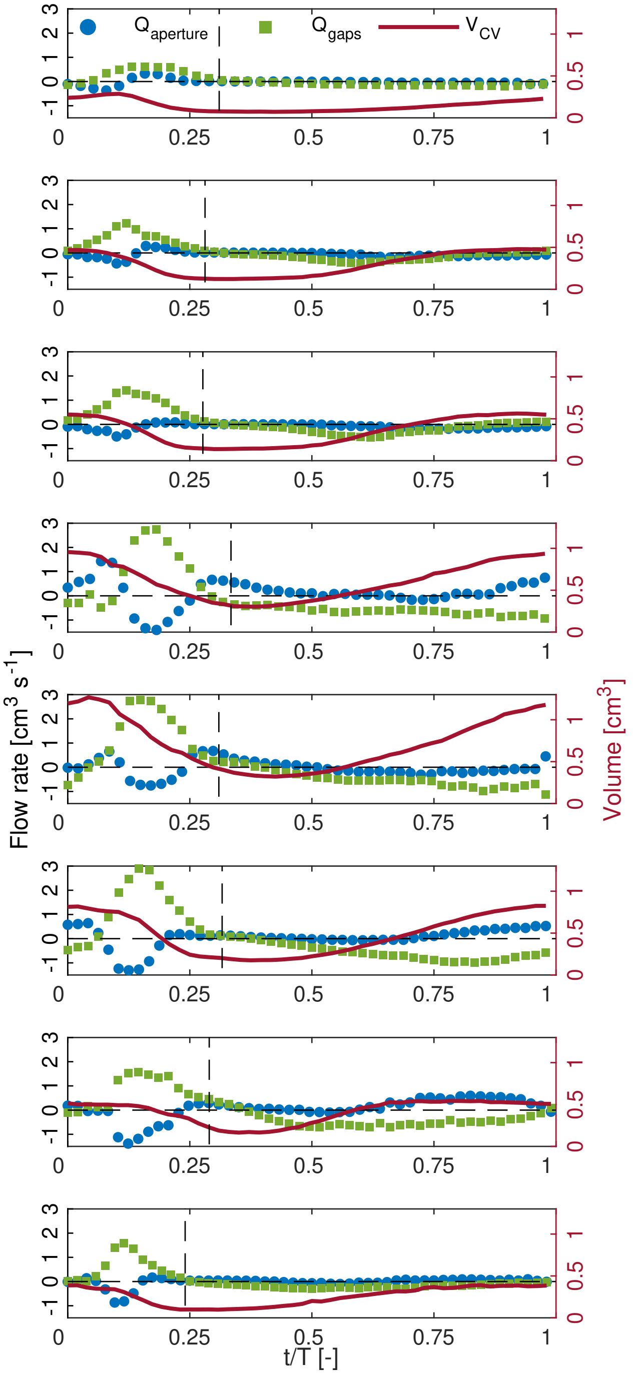

Fig. S3. Phase-averaged flow rates in the eight polyps studied. Each panel shows the phase-averaged flow rates through the top aperture of the polyp (blue circles) and through the gaps (green squares). The red line represents the volume encapsulated by the polyp (right y-axis). See Table S1 for the conditions in each measurement, where the row in the table corresponds to the row in the figure.

Journal of Experimental Biology • Supplementary information

Table S1. Conditions in the PIV experiments (A) and Lagrangian analyses (B).

(A) Conditions in PIV experiments.

	Duration	$N_{pulsations}$	T	T_{up}	T_{down}	Domain length	Domain width	D_{polyp}
	[s]	[#]	[s]	[s]	[s]	[cm]	[cm]	[cm]
1	44.4	26	1.71	0.53	1.17	3.34	2.05	1.41
2	49.3	22	2.24	0.63	1.61	3.36	2.07	1.59
3	49.3	22	2.24	0.62	1.61	3.58	2.20	1.82
4	35.5	18	1.97	0.66	1.41	4.02	2.47	2.03
5	69.3	33	2.10	0.65	1.44	3.47	2.14	2.05
6	47.3	22	2.15	0.68	1.46	3.96	2.46	1.86
7	52.2	24	2.17	0.63	1.54	3.87	2.38	1.54
8	55.9	24	2.33	0.56	1.76	3.06	1.88	1.29

(B) Conditions in Lagrangian Analysis. T_{PSS} is the time when the number of tracers entering the domain roughly equaled the number of tracers exiting it, and T_{Total} is the duration of the Lagrangian analysis after stitching to 50 pulsation periods. β represents the angle of inclination of the polyp relative to the video frame. V_{domain} represents the volume of the domain Ω_{domain}

	V_{domain}	D_{polyp}	$\frac{V_{domain}}{D_{polyp}}$	C_0	β	T_{PSS}	T_{total}	$\frac{T_{PSS}}{T_{total}}$
	cm^3	cm	cm^2	$\frac{Tracers}{cm^3}$	$\circ$	s	s	$-$
1	23.7	1.41	16.9	1266	-3.8	38.0	86.7	0.44
2	10.7	1.59	6.7	559	11.6	85.4	113.0	0.75
3	12.65	1.82	6.9	462	12.5	85.7	113.2	0.76
4	33.1	2.03	16.3	309	12.3	47.3	96.2	0.49
5	17.8	2.01	8.7	1248	-4.1	77.9	107.9	0.72
6	19.0	1.86	10.2	218	18.2	77.0	108.8	0.71
7	23.4	1.54	16.4	1023	9.2	28.1	110.5	0.25
8	13.1	1.29	10.2	643	13.7	61.4	118.4	0.52

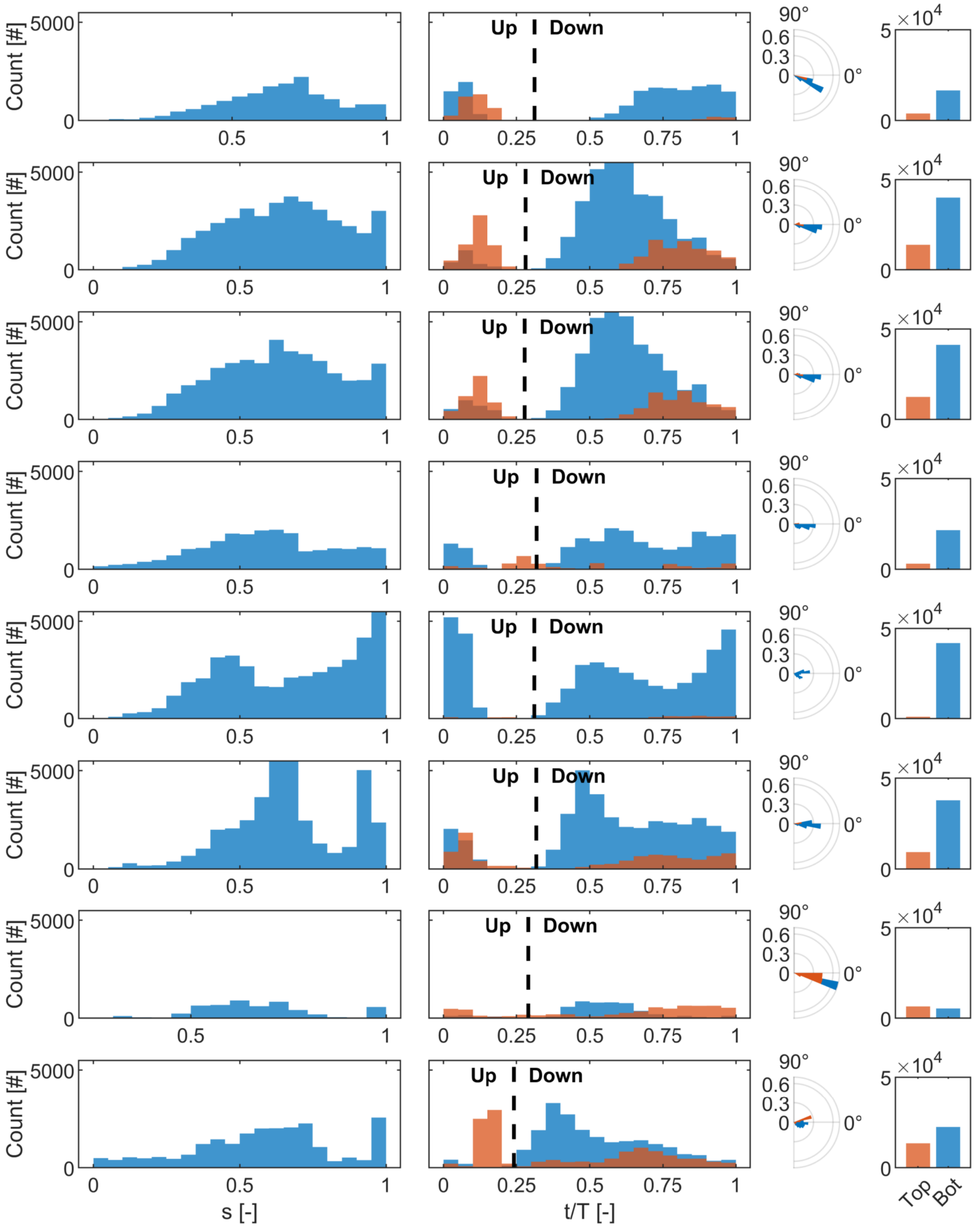

Fig. S4. Distribution of water parcel interception in eight polyps. The histograms display the spatial (first column) and temporal (second column) distributions of water parcel interception, the direction of origin of tracers that intercept the polyp (third column), and the amount of tracers interception with the oral and aboral sides of the polyp (fourth column). The blue bars indicate tracers contacting the aboral surface (Top), and the orange bars indicate tracers contacting the oral surface (Bot). Areas where these bars overlap appear brown. Each row represents one polyp out of the eight displayed aggregated in Fig. 8 and separately in Fig. S4. Note that polar histograms displays proportion of tracers, and other histograms display count. Each row in this figure represents a specific Lagrangian analysis, as detailed in Table S1 B, with each entry in the table corresponding to the rows shown here.

Journal of Experimental Biology • Supplementary information

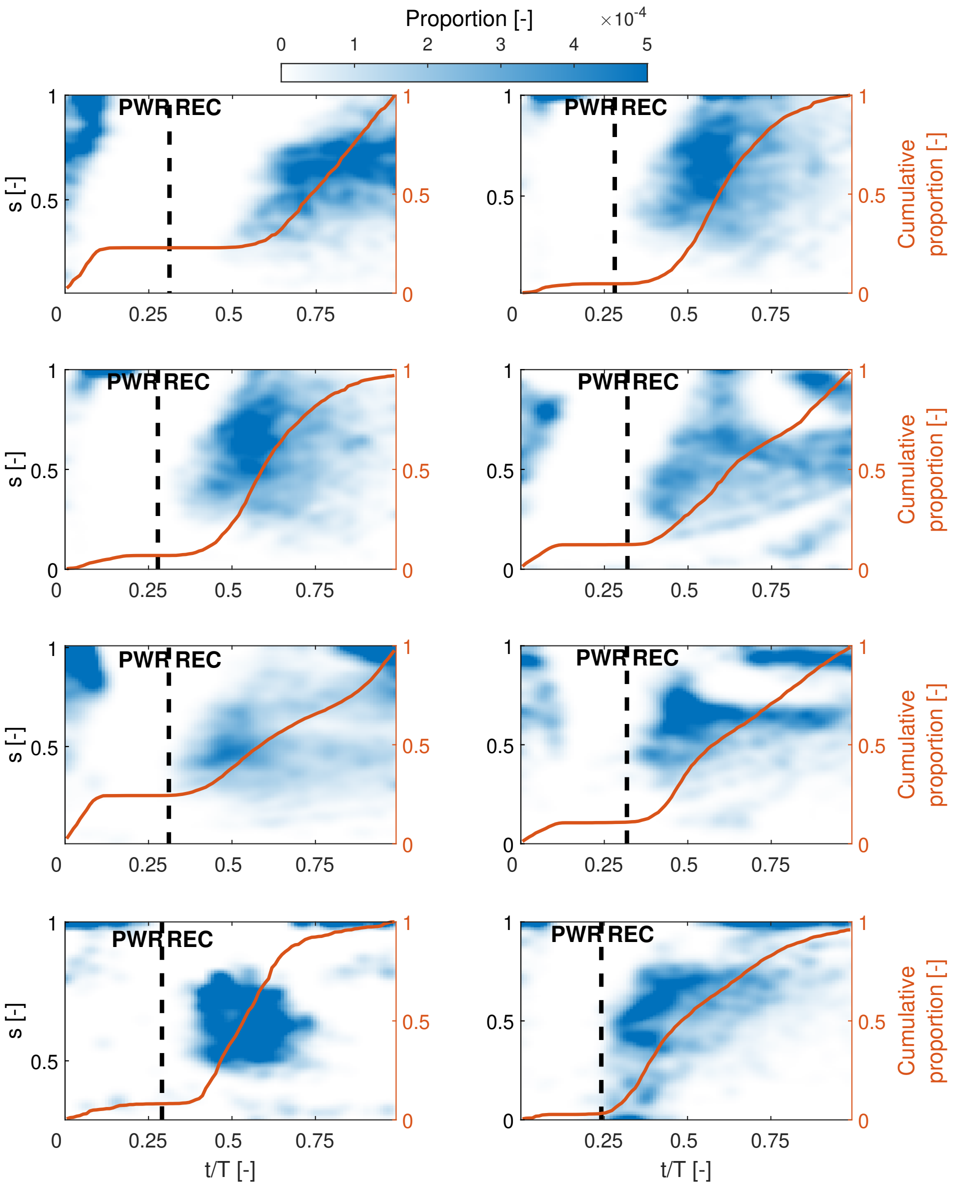

Fig. S5. Spatio-temporal distribution of water parcel interception. Each panel presents the spatio-temporal patterns of water parcel capture in each of the eight polyps show aggregated in Fig. 8. The x-axis is time and the y-axis is the legnth along the tentacles, where 0 is the tentacle base and 1 is the tentalcle tip. The color map represents the proportion of water parcel interception proportion (see color bar at the top). The spatio-temporal patterns of water parcel interception are similar in the eight polyps, where the tip of the tentacle experienced a peak in the proportion of interception at the beginning of the upward stroke and the end of the downward stroke, and the middle of the tentacle experiences its peak a bit after the downward stroke begins. The boundary between the upward (PWR) and downward (REC) strokes is marked with a dashed horizontal line. The orange line (right y-axis) represents the cumulative proportion of water parcel collision with the polyp as a function of time. Each panel in this figure represents a specific Lagrangian analysis, as detailed in Table S1B, with each entry in the table corresponding to the panels shown here, arranged left to right, top to bottom.

Journal of Experimental Biology • Supplementary information

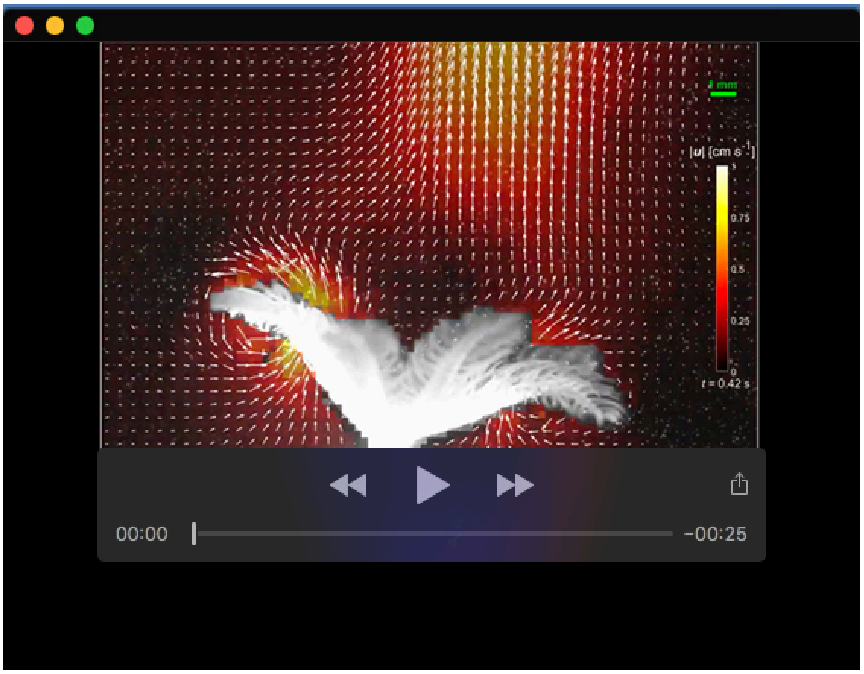

Movie 1. The flow field during two pulsation periods. The quiver arrows represent point measurements of the flow field. For presentation reasons only 25% of the vectors measured are shown. The heatmap overlay represents the velocity magnitude $|\boldsymbol{u}|$ (see colorbar for scale). The video is slowed down by a factor of 3.5. Click the image to acess the video on YouTube.

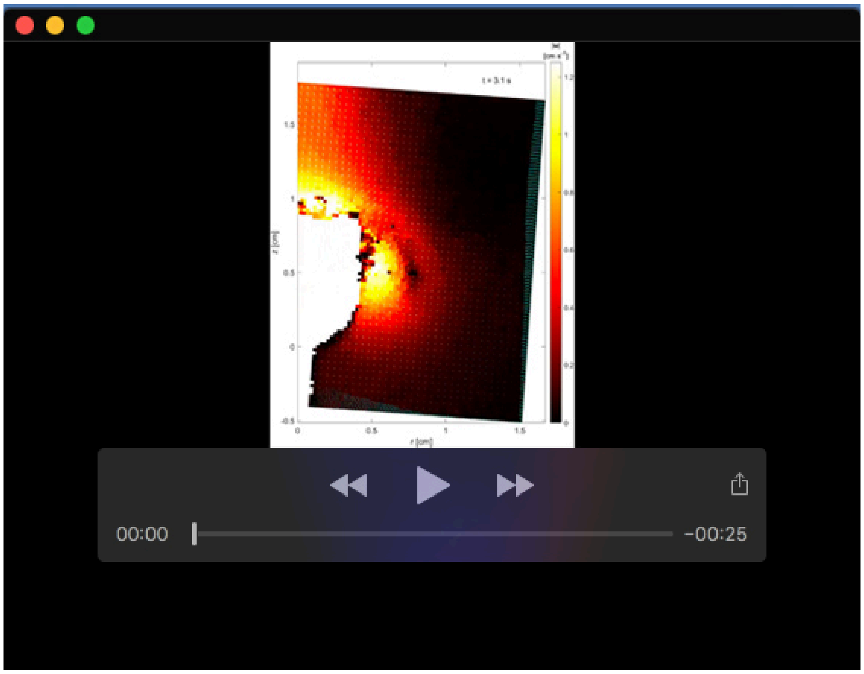

Movie 2. Trajectories of imaginary inertia-less tracers. Tracers (cyan dots) are introduced from the boundaries of the domain at the same concentration C_0, move with the measured flow field, and disappear from the simulation when they encounter the polyp or exit through the top. The quiver arrows represent point measurements of the flow field. For presentation reasons only 25% of the vectors measured are shown. The heatmap overlay represents the velocity magnitude $|\boldsymbol{u}|$ (see colorbar for scale). The video is sped up by ˜30%. Click the image to acess the video on YouTube.

Journal of Experimental Biology • Supplementary information
